# Remodelling of lace plant leaves: antioxidants and ROS are key regulators of programmed cell death

**DOI:** 10.1007/s00425-017-2683-y

**Published:** 2017-04-07

**Authors:** Adrian N. Dauphinee, Jacob I. Fletcher, Georgia L. Denbigh, Christian R. Lacroix, Arunika H. L. A. N. Gunawardena

**Affiliations:** 1 Department of Biology, Dalhousie University, 1355 Oxford Street, Halifax, NS B3H 4R2 Canada; 2 Department of Biology, University of Prince Edward Island, 550 University Ave, Charlottetown, PE C1A 4P Canada

**Keywords:** Anthocyanins, Antioxidant enzymes, Live cell imaging, Perforation development, Reactive oxygen species, Spectrophotometry

## Abstract

**Antioxidants and reactive oxygen species are integral for programmed cell death signaling during perforation formation in the lace plant (** ***Aponogeton madagascariensis*** **).**

The lace plant is an excellent model system for studying developmentally regulated programmed cell death (PCD). During early lace plant leaf development, PCD systematically deletes cells resulting in a perforated leaf morphology that is unique *in planta.* A distinct feature in young lace plant leaves is an abundance of anthocyanins, which have antioxidant properties. The first sign of PCD induction is the loss of anthocyanin pigmentation in cells that are targeted for destruction, which results in a visible gradient of cell death. The cellular dynamics and time course of lace plant PCD are well documented; however, the signals involved in the pathway remain elusive. This study investigates the roles of antioxidants and ROS in developmental PCD signaling during lace plant perforation formation. The involvement of antioxidants and ROS in the pathway was determined using a variety of techniques including pharmacological whole plant experimentation, long-term live cell imaging, the 2,2′-azino-bis-3-ethylbenzothiazoline-6-sulfonic acid anti-radical activity assay, and western blot analysis. Results indicate that antioxidants and ROS are key regulators of PCD during the remodelling of lace plant leaves.

**Electronic supplementary material:**

The online version of this article (doi:10.1007/s00425-017-2683-y) contains supplementary material, which is available to authorized users.

## Introduction

### Programmed cell death

Programmed cell death (PCD) is a series of tightly controlled events leading to the demise of targeted cells (Kacprzyk et al. 2011; Bozhkov and Lam 2011). In multicellular eukaryotes, it occurs as part of normal development or in the maintenance of tissue homeostasis and, therefore, is a critical mechanism for survival (Coll et al. 2011). The signaling cascades of animal PCD are well understood in comparison to plants and have clearly defined molecular subroutines, as described by the nomenclature committee on cell death (Kroemer et al. 2005, 2009; Galluzzi et al. 2012). The identification of key regulators of plant PCD has been the focus of recent studies, which will contribute to our understanding of the process and the development of robust classification systems (van Doorn et al. 2011; Dauphinee and Gunawardena 2015).

### Reactive oxygen species in PCD

Reactive oxygen species (ROS) are chemically unstable oxygen derivatives that act as signaling molecules in aerobic organisms for several biological processes in development, growth, and responses to environmental stimuli (Gechev et al. 2006; Baxter et al. 2014; Petrov et al. 2015). Elevated levels of ROS such as hydrogen peroxide (H_2_O_2_), superoxide (O_2_^−^), and reactive nitrogen species including nitric oxide (NO) are associated with PCD. ROS influence the production of phytohormones including ethylene, jasmonic acid, and salicylic acid, or cause posttranslational modifications that ultimately activate the genes, proteases, and nucleases that carry out PCD (Van Breusegem and Dat 2006). In plants, major sources for ROS production are mitochondria, chloroplasts, peroxisomes, and cell walls through the activity of class III cell wall peroxidases and NADPH oxidases (Mignolet-Spruyt et al. 2016). The accumulation of ROS within cells can also trigger production of ROS-scavenging antioxidants, including but not limited to: anthocyanins, glutathione, ascorbic acid, superoxide dismutase 1 (SOD1), catalase (CAT), and glutathione peroxidase (Pandhair and Sekhon 2006; Ahmad et al. 2010). Although ROS have long been viewed as strictly detrimental compounds, they are now known to play important roles during normal cell signaling and homeostasis. The roles of redox homeostasis in the perception, signaling, and physiological responses in plants have been studied extensively (see Pavet et al. 2005; Mignolet-Spruyt et al. 2016).

### The lace plant model system

**Fig. 1:**
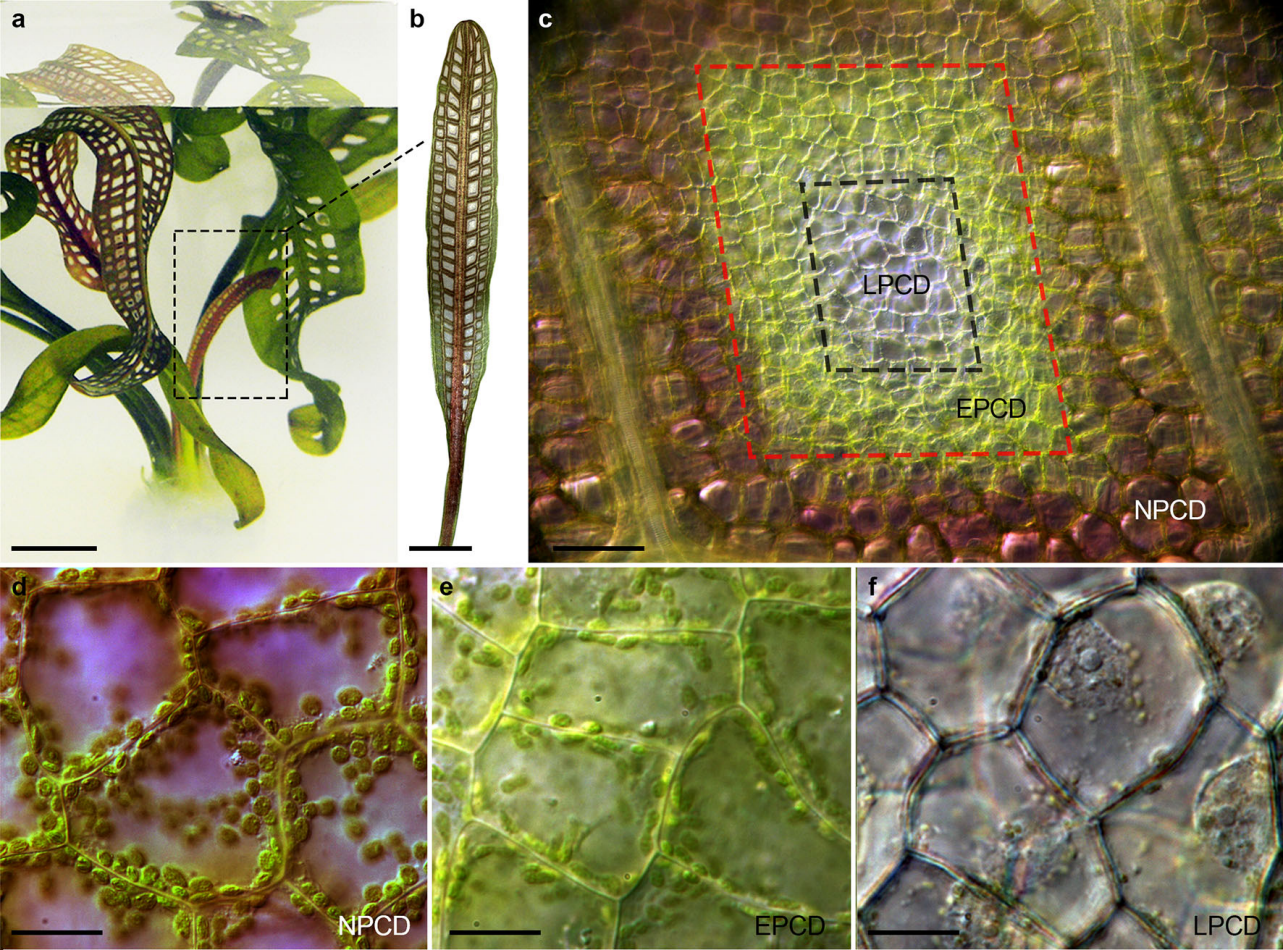
Lace plant (*A. madagascariensis*) model system. **a** Axenic lace plant cultures are maintained in Magenta GA-7 boxes. **b** Programmed cell death (PCD) is actively occurring in the window stage of leaf development (*dashed line*, **a**) between longitudinal and transverse veins (**c**) where a unique gradient of cell death exists as highlighted by the *dashed lines*. **d** Non-PCD (NPCD) cells are replete with anthocyanins and do not undergo PCD during leaf morphogenesis. **e** Early programmed PCD (EPCD) cells are fated to die and have lost their anthocyanin pigmentation. **f** Late-programmed PCD (LPCD) cells are nearly transparent and are on the verge of death. *Bars* 1 cm (**a**), 5 mm (**b**), 100 µm (**c**), and 25 µm (**d**–**f**)

The lace plant (*Aponogeton madagascariensis*) is an aquatic monocot that forms a unique perforated leaf morphology by the removal of specific cells via developmentally regulated PCD (Fig. 1a; Gunawardena et al. 2004). The process of perforation formation has been characterized into five stages of leaf development by Gunawardena et al. (2004). Young leaves in the pre-perforation stage emerge from the corm with a complete vein pattern but are tightly furled and have a red pigmentation due to the presence of anthocyanins. Anthocyanins scavenge a wide array of reactive oxygen and nitrogen species; in fact, purified anthocyanins can be up to four times more efficient than ascorbate and α-tocopherol (Wang et al. 1997; Gould 2004). The window stage is distinguishable in the unfurled leaves which have populations of central cells actively undergoing PCD within the areoles located between longitudinal and transverse veins (Fig. 1b). Areoles of window stage leaves (Fig. 1c) exhibit a distinct gradient of PCD, as described by Lord et al. (2011). Non-PCD or NPCD stage cells (Fig. 1d) contain anthocyanins and do not die during perforation formation. Early programmed PCD (EPCD; Fig. 1e) cells have lost anthocyanin coloration and are fated to die. Late-programmed PCD (LPCD; Fig. 1f) cells are nearly transparent and on the brink of death. Following the window stage is perforation formation, where PCD advances and cells in the centermost portion of the areole are removed. The perforation increases in size during the expansion phase before halting 4–5 cell layers from the veins by the mature stage. Mesophyll cells at the perforation border then transdifferentiate into epidermal cells and deposit a protective layer of suberin (Gunawardena et al. 2007).

The lace plant has emerged as a model system for studying PCD due to the spatial and temporal predictability of PCD in leaves, which are thin and nearly transparent making them ideal for microscopy, as well as established axenic cultures suitable for pharmacological experiments (Gunawardena et al. 2006). The conspicuous pattern and disappearance of anthocyanins during the window stage in cells undergoing PCD during lace plant perforation formation suggests that antioxidant levels and ROS could be involved in this signaling pathway. It is hypothesized that antioxidants and ROS play a significant role in the induction of lace plant PCD. This study employed whole plant experimentation, live cell imaging, spectrophotometric assays, and western blot analysis to elucidate the effects of antioxidants and ROS on lace plant PCD. Data indicate that antioxidants and ROS are key regulators of lace plant developmental PCD signaling.

## Materials and methods

### Tissue culturing and whole plant experiments

*Aponogeton madagascariensis* (Mirbel) H. Bruggen cultures were established and propagated according to Gunawardena et al. (2006). Plants were grown in Magenta GA-7 vessels containing 50 ml of solid MS medium consisting of 1.5% agar (Phytotechnology Laboratories) and 150 ml of liquid MS. Cultures were kept at 24 °C under daylight deluxe fluorescent light bulbs (Philips) at an intensity of 125 μmol/m^2^/s on 12 h light/dark cycles. Whole plant experiments were carried out using plants with a minimum of three perforated mature leaves. Optimized treatments included: (1) an antioxidant treatment of 400 µg/ml l-ascorbic acid and 200 µg/ml l-cysteine (Bioshop Canada), (2) a ROS treatment of 1 mM H_2_O_2_ (Fisher Scientific), and (3) the antioxidant + ROS treatment which consisted of a treatment with the antioxidants, followed by 1 mM H_2_O_2_ 4–5 days later. Plants were allowed to grow for 2 weeks before their leaves were harvested and analyzed. Leaf measurements included length, width, and the number of perforations formed. A minimum of 12 plants were treated for each group.

### Long-term live cell imaging

Time-lapse videos were captured using the audio–video interleave (AVI) function of Nikon NIS AR software controlling a Nikon Eclipse 90*i* compound light microscope (Nikon Canada Inc). The live cell imaging technique described by Wertman et al. (2012) was employed, with some modifications. Whole window stage leaves were removed from plant cultures, rinsed with distilled water, and mounted in a custom grooved slide that matched the width and depth of the leaf midrib and allowed the blade to lie flat on the slide surface. Depending on the experimental conditions, either distilled water or a treatment was applied to the leaf before a glass coverslip was applied and sealed with melted VALAP (2:1:1, w/w mixture of paraffin wax, vaseline, and lanolin). Leaf observations were carried out for 12 h daily. The leaf was rinsed with distilled water and remounted every 6 h to reduce stress and contamination, and kept in the dark at 24 °C in fresh media overnight until the next period of imaging. The time of death was determined at the point where all PCD area cells up to the NPCD boundary had expired. A minimum of three independent replicates were carried out for each treatment group.

### Nitro blue tetrazolium (NBT) staining

Histochemical detection of O_2_^−^ in window stage leaves was performed using a modified protocol from Grellet Bournonville and Díaz-Ricci (2011). Leaves from the various treatment groups were cut into 5 mm^2^ sections and then immersed in stain solution consisting of 50 mM potassium phosphate buffer (7.8 pH), 10 mM sodium azide, and 0.1% NBT (Sigma-Aldrich). The samples were then kept in the dark, vacuum infiltrated at 15 psi for 15 min, and then incubated at room temperature for 15 min prior to microscopic observation. The negative control underwent the same procedure with the solution lacking NBT. After staining, specimens were mounted in distilled water and viewed using a Nikon Eclipse Ti microscope. To confirm the observed staining patterns in the various leaf stages without the interference of anthocyanin and chlorophyll pigmentation, samples were placed in 95% ethanol for 2–3 days. A minimum of three replicates were carried out for all groups.

### Detection of superoxide dismutase-1 (SOD1) and catalase (CAT)

Harvested leaves had their midrib removed, blot-dried, and frozen with liquid nitrogen. The tissues were homogenized on ice in a 1:1 ratio of Pipes buffer (6.8 pH) to protease inhibitor solution. The protease inhibitor solution was a 1:2 ratio of component A to component B, respectively. Component A was comprised of 10 mg/ml leupeptin and 10 mg/ml soybean trypsin inhibitor (Sigma-Aldrich) dissolved in Pipes buffer. Component B consisted of 10 mg/ml pepstatin and 20 mg/ml PMSF dissolved in 95% ethanol. The homogenized samples were then centrifuged at 16,000*g* for 15 min. The supernatants were removed and quantified for total protein concentration via Bradford assay (Bradford 1976). Sample preparations for SDS-PAGE were made using a 1:1 ratio of sample to 2× Laemmli sample buffer (Bio-Rad) with 5% β-mercaptoethanol (v/v). The Precision Plus Protein Standards (Bio-Rad) and samples (10 μg protein weight) were loaded into 8–16% SDS polyacrylamide Mini-PROTEAN TGX precast gels (Bio-Rad) and resolved at 160 V for 1 h in ice cold running buffer (0.1% SDS (v/v), 25 mM Tris and 192 mM glycine, 8.3 pH). Proteins were transferred overnight at 120 mA to a 0.2 µm nitrocellulose membrane (Bio-Rad) in transfer buffer (20% methanol (v/v), 25 mM Tris, and 192 mM glycine, 8.3 pH) at room temperature.

Nitrocellulose membranes were blocked for 1 h at room temperature with mild shaking using 3% (w/v) low fat milk powder in TBS-T (Tris buffered saline with Tween 20; 10 mM Tris, 140 mM NaCl, and 0.1% Tween-20, 7.4 pH). The membrane was then incubated at room temperature for 30 min in a 1:1000 dilution of the SOD1 rabbit polyclonal antibody (Santa Cruz Biotechnology, #sc-11407) in TBS-T, and then rinsed four times for 1, 1, 2, and 3 min, respectively. The membrane was then transferred to TBS-T with a 1:10,000 dilution of goat anti-rabbit IgG HRP-conjugated antibody (Santa Cruz Biotechnology, #sc-2004) for 30 min, and then rinsed as mentioned above with the addition of a final 2 min rinse in TBS. Secondary antibody localization was achieved using a Pierce ECL Western Blotting Substrate (Thermo Fisher Scientific) according to the manufacturer’s instructions and imaged using a MF-ChemiBIS 3.2 gel documentation system (DNR Bio-Imaging). Following imaging, the membrane was rinsed for 5 min in TBS-T and then incubated overnight at 2 °C in a 1:5000 dilution of CAT rabbit polyclonal antibody (Agrisera, #AS09 501) in 5% (w/v) low fat milk in TBS-T. The next day, the secondary antibody incubation and imaging was performed as mentioned above. A minimum of four independent replicates were carried out for all treatment groups.

Protein band intensities were normalized to their respective control via Ponceau staining, which served as the loading control. Ponceau staining was done for 5 min with mild shaking at room temperature, followed by a 2 min rinse with TBS-T. An image of the Ponceau-stained membrane was converted to greyscale and imported into Image Studio Lite and quantified (Li-Cor Biosciences). Image Studio Lite was also used to determine individual band intensities.

### Anthocyanin and ABTS spectrophotometric assays

Tissue samples (20 mg) were excised from mature and window stage leaves taken from sterile cultures. The anthocyanin extraction protocol was adapted from Li et al. (2010). Tissue samples were ground and macerated in 200 μl of formic acid/methanol (5/95, v/v) and placed at room temperature in the dark for 50 min, followed by 10 min centrifugation at 10,000*g*. The supernatant was collected and absorbance immediately read at 520 nm using a SmartSpec Plus Spectrophotometer (Bio-Rad). The 2,2′-azino-bis-3-ethylbenzothiazoline-6-sulphonic acid (ABTS) assay kit was used according to the manufacturer’s instructions (Zen-Bio). Absorbance was determined using a SpectraMax Plus 384 Microplate Reader and Softmax Pro 5 software (Molecular Devices). Standard curves of ascorbic acid (ABTS) and cyanidin-3-rutinoside (anthocyanin) were generated, and results were expressed as vitamin C equivalents and cyanidin-3-rutinoside equivalents (C3RE), respectively. A minimum of 4 replicates were analyzed for each group.

### Image and video processing

Photographs were acquired using a Nikon L110 digital camera. All images and videos were prepared for publication using Photoshop and Premiere Pro, respectively (Adobe Creative Cloud; Adobe Systems Inc.). When necessary to improve image quality, alterations to brightness, contrast, and color were made evenly. In the whole plant layouts and detached window stage leaf images, backgrounds and or shadows were removed using Photoshop. Window stage leaf micrographs were acquired by a Nikon AZ100 microscope and multiple images were merged together using the layer mask tool in Photoshop.

### Statistical analysis and data representation

The one-way ANOVA followed by a Tukey or Dunnett’s test was carried out to detect significant differences among means. All data are expressed as mean ± SE unless otherwise stated. Analyses were carried out using the GraphPad Prism 5 software (GraphPad Software Inc.).

## Results

### Whole plant experiments

**Fig. 2:**
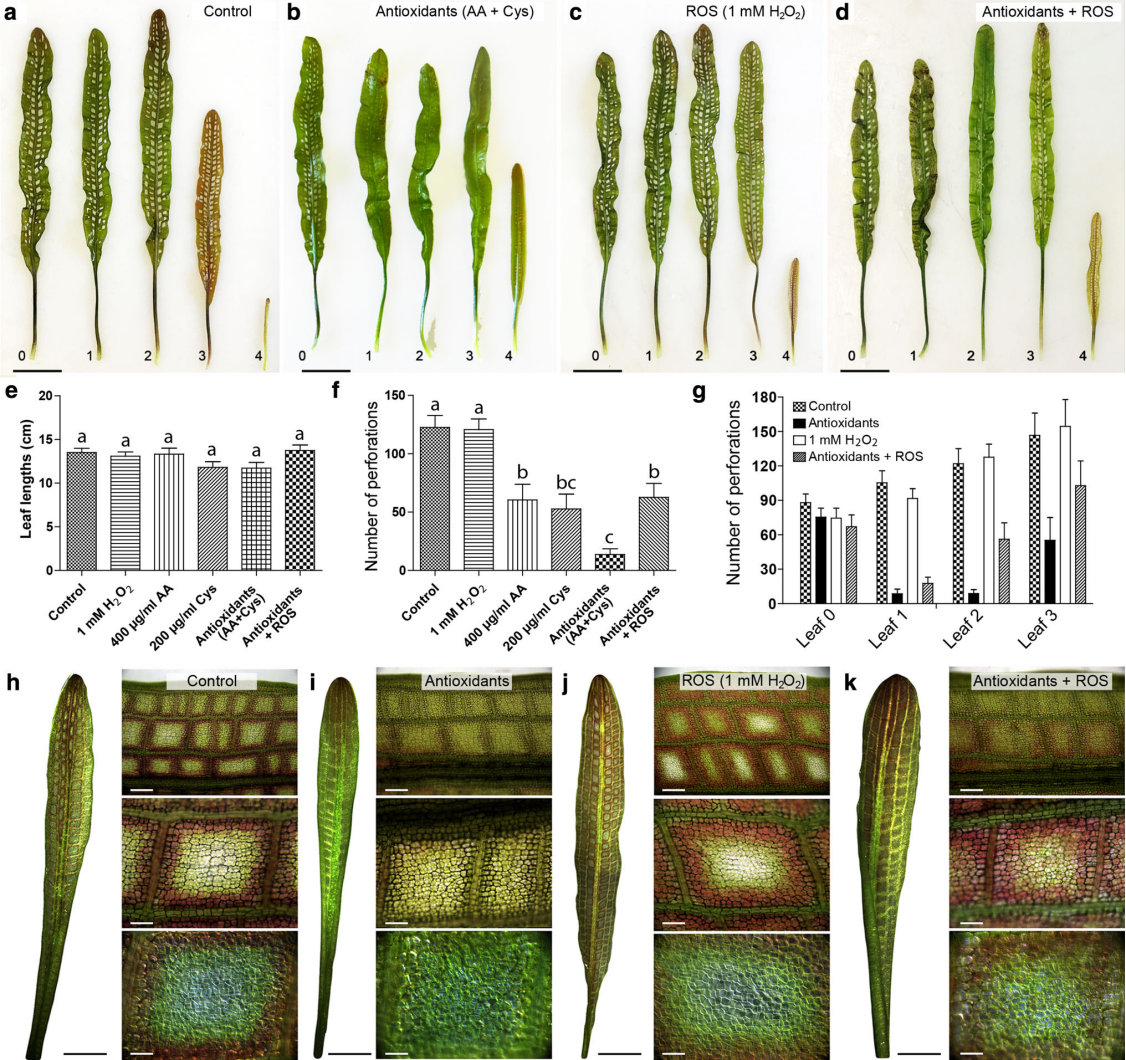
Whole plant effects of antioxidant and ROS treatments. Representative leaf layouts showing the control (**a**), the antioxidant combination of 400 µg/ml ascorbic acid (AA) and 200 µg/ml cysteine (Cys) (**b**), 1 mM H_2_O_2_ and an antioxidant + ROS treatment receiving 400 µg/ml AA (**c**), and 200 µg/ml Cys followed by 1 mM H_2_O_2_ 4–5 days later (**d**). Leaves were arranged chronologically—leaf 0 represents a control leaf, which developed prior to treatment, while leaves 1–4 developed afterwards (4 being the youngest). The length (**e**) and the mean number of perforations (**f**) were quantified for post-treatment mature leaves along with the mean number of perforations by leaf (**g**). Representative window stage images (1 week after the beginning of the experiment) were taken for control (**h**), the AA and Cys combination (**i**), 1 mM H_2_O_2_ (**j**) and antioxidant + ROS (**k**) treatments. *Bars* 2 cm (**a**–**d**), 5 µm (leaves, **h**–**k**), 250, 100, and 50 µm (micrographs, *top* to *bottom*, **h**–**k**). Means with different letters differ significantly and *error bars* represent standard error. One-way ANOVA, Tukey test (*P* < 0.05; *n* ≥ 12)

Lace plants grown in axenic cultures were utilized to determine the effects of antioxidants and ROS on developmental PCD and perforation formation. Control plants (Fig. 2a) had mature leaves with a length of 13.51 ± 0.49 cm (Fig. 2e) and 122.60 ± 10.26 perforations (Fig. 2f). An optimized antioxidant combination treatment of 400 µg/ml ascorbic acid (AA) and 200 µg/ml l-cysteine (Cys; Fig. 2b) resulted in leaves with a length of 13.09 ± 0.49 cm (Fig. 2e) and 13.67 ± 4.92 perforations (Fig. 2f), which was significantly fewer perforations than controls. A 1 mM H_2_O_2_ (Fig. 2c) ROS treatment showed no differences from the control in terms of leaf length (13.09 ± 0.49 cm; Fig. 2e) and perforations (120.70 ± 9.11; Fig. 2f). Higher concentrations of H_2_O_2_ (≥5 mM) were too extreme and killed the plants’ leaves (data not shown). The antioxidant + ROS treatment (Fig. 2d) resulted in significantly fewer perforations (62.69 ± 11.83; Fig. 2f) than the control, but more than the antioxidant [ascorbic acid (AA) + cysteine (Cys)] treatment alone. The antioxidant + ROS treatment group leaf lengths of 13.74 ± 0.6254 cm (Fig. 2e) did not differ significantly from the control.

The number of perforations by leaf was analyzed for the control, antioxidant, ROS, and antioxidants + ROS groups (Fig. 2g). Control and 1 mM H_2_O_2_ treated plants showed very similar trends with a steady increase in the number of perforations in each subsequent leaf (Fig. 2g). The antioxidant and the antioxidant + ROS treatment groups both had strong PCD inhibition by leaf 1; however, perforation formation sharply increased in the antioxidant + ROS treatment by leaves 2 and 3 (Fig. 2g). Micrographs of window stage leaves (Fig. 2h–k), 1 week following the onset of experimentation, showed a visible difference in anthocyanin pigmentation in the ascorbic acid (AA) + cysteine (Cys) treatment group (Fig. 2i), which did not have the typical window stage gradient of cell death as seen in the control (Fig. 2h) and H_2_O_2_ specimens (Fig. 2j). The window stage leaves of the antioxidant + ROS treatment (Fig. 2k) had anthocyanin pigmentation, but it was not as prominent as the control and H_2_O_2_ leaves.

### ROS detection in lace plant PCD

**Fig. 3:**
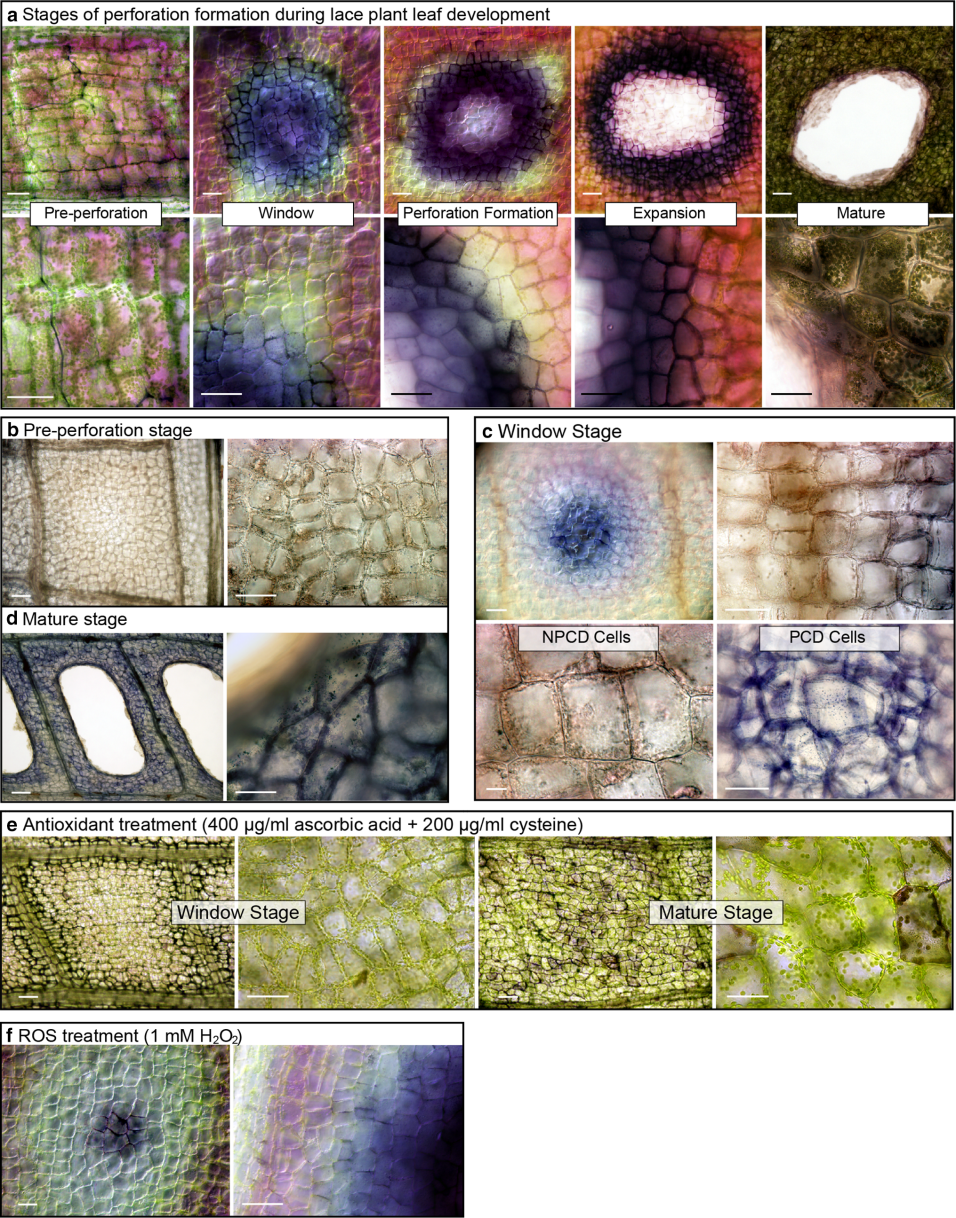
Superoxide detection using nitro blue tetrazolium (NBT) staining in lace plant leaves. Pre-perforation stage leaves in which programmed cell death (PCD) has not yet initiated (**a**). Window stage leaves, where PCD is actively occurring in the center of areoles. PCD radiates outward and a hole forms centrally in the perforation formation stage. The perforation expansion is demarcated by a drastic increase of dead cells and enlargement of the hole, and precedes the mature stage where PCD halts 4–5 cell layers from the veins. Cleared and fixed stained specimens for the pre-perforation (**b**), window (**c**), and mature (**d**) stages. Antioxidant (400 µg/ml ascorbic acid + 200 µg/ml cysteine) treated window and mature stage leaves (**e**). Early window stage leaf treated with ROS (1 mM H_2_O_2_) (**f**). *Bars* 40 µm

The stages of lace plant perforation formation were investigated in terms of ROS production, specifically O_2_^−^ using nitro blue tetrazolium (NBT) staining (Fig. 3a). Pre-perforation stage leaves showed little to no NBT staining. Window stage leaves had NBT staining predominantly in PCD cells. As the perforation formed centrally, the most abundant staining was in the late-programmed cell death stage (LPCD). Dark staining in PCD cells bordering the degraded cells was also observed during the expansion phase of the perforation. In mature leaves, light NBT staining was visible but ubiquitous throughout the cells (Fig. 3a). Specimens fixed and cleared in 95% ethanol were also subjected to NBT to confirm the staining patterns without obfuscation from the naturally occurring leaf pigments. Likewise, NBT staining was observed in all stages; most importantly, there was little to no staining in pre-perforation leaves (Fig. 3b), or the NPCD region of the window stage (Fig. 3c). PCD cells of the window stage (Fig. 3c) were prominently stained and there was ubiquitous staining in the mature stage (Fig. 3d). NBT staining of antioxidant-treated window stage leaves had little to no formazan precipitation, while the mature leaves had some staining throughout the tissue (Fig. 3e). Leaves treated with ROS (1 mM H_2_O_2_, Fig. 3f) had similar staining to the controls.

### Long-term live cell imaging

**Fig. 4:**
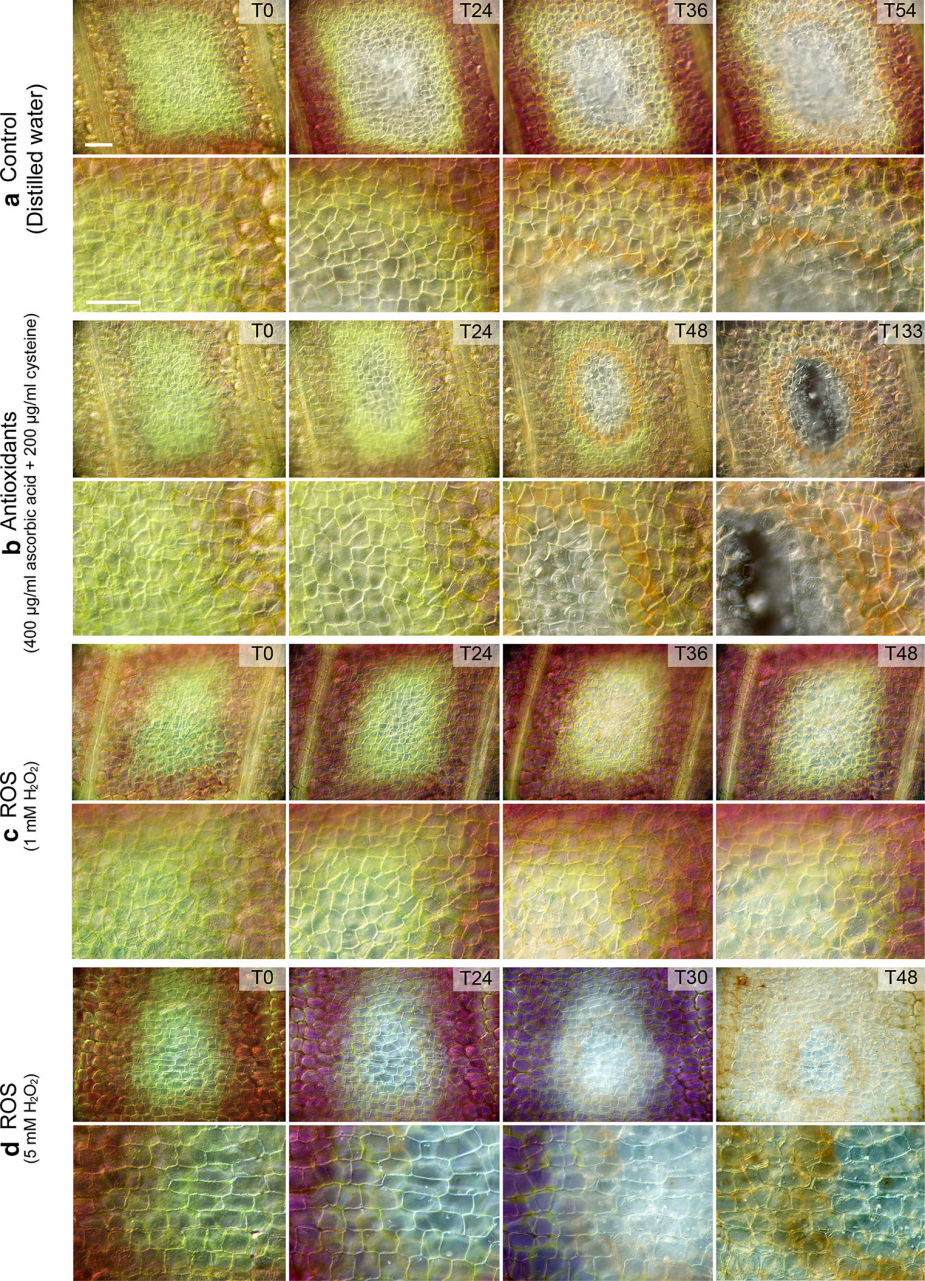
Long-term live cell imaging of window stage leaves. Control leaves at T0, 24, 36, and 54 h, just prior to the death of the leaf (**a**). Antioxidant-treated leaves [400 µg/ml ascorbic acid (AA) and 200 µg/ml l-cysteine (Cys)] at T0, 24, 48, and 133 h (**b**). 1 mM H_2_O_2_ ROS treatment at T0, 24, 36, and 48 h (**c**). The 5 mM H_2_O_2_ treatment at T0, 24, 30, and 48 h (**d**). The *top row* shows a generalized view of the areole, the *bottom* focuses on the PCD gradient at a higher magnification. Please see Online Resource S1 and S2 for more detail. *Bars* 50 µm

**Fig. 5:**
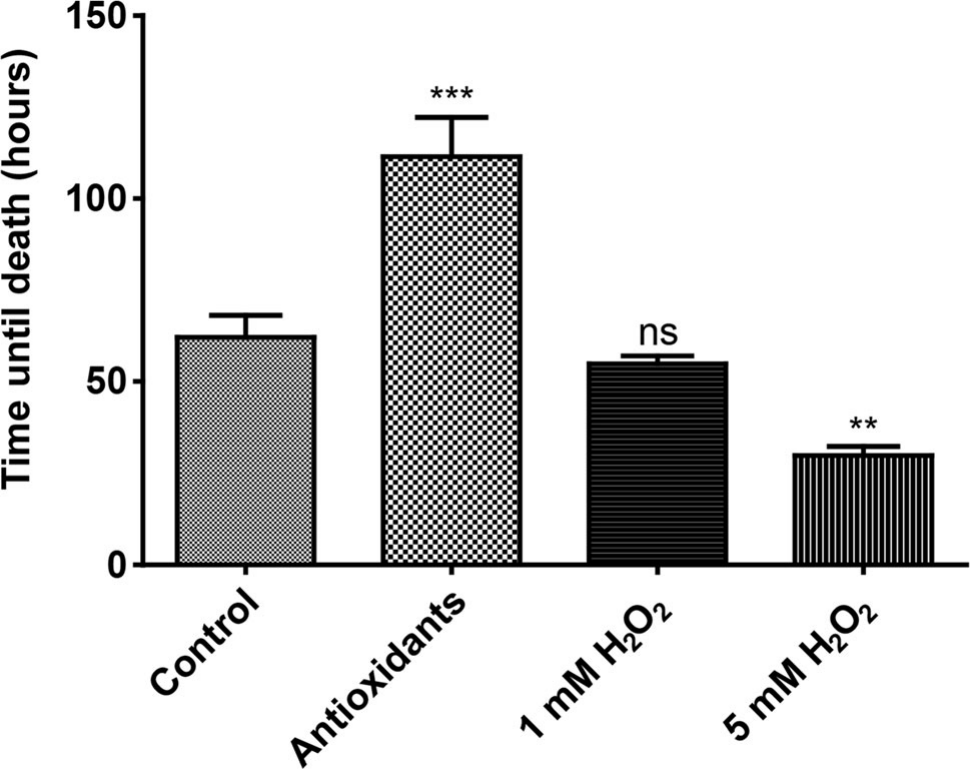
Long-term live cell imaging, mean times for death of PCD areas. Window stage leaves were observed under the control, antioxidant (400 µg/ml ascorbic acid + 200 µg/ml cysteine), 1 mM H_2_O_2_ and 5 mM H_2_O_2_ treatment groups. Antioxidant treatment significantly increased the mean time for death compared to the control, while the 1 mM H_2_O_2_ treatment had no effect; however, 5 mM H_2_O_2_ exposure increased the rate of death (one-way ANOVA, Dunnett’s multiple comparison test, ***P < 0.001; **P < 0.01; ns, non-significant; *n* = 3). *Error bars* represent standard error

The treatments optimized in whole plant experiments were utilized for detached, early window stage leaves. The detached leaves were observed using a modified long-term live cell imaging technique developed by Wertman et al. (2012) (Fig. 4; Online Resource S1 and S2). In the control treatment (Fig. 4a), the time for cell death was 62.08 ± 4.89 h (Fig. 5). The antioxidant treatment (Fig. 4b) slowed the progression of PCD significantly compared to the control, as the time for death was 111.43 ± 8.80 h (Fig. 5). There was also a strong phenolic ring that formed centrally, approximately 36–48 h into the observation (T48; Fig. 4b), which was more pronounced than that observed in control samples (T36; Fig. 4a). The 1 mM H_2_O_2_ ROS treatment (Fig. 4c) had a time of death of 54.85 ± 1.75 h (Fig. 5), which did not differ significantly from the control. The 5 mM H_2_O_2_ ROS treatment increased the rate of cell death significantly (Fig. 4d), as the time of death was 29.88 ± 2.21 h (Fig. 5). After death of the PCD area, even NPCD cells lost their pigmentation and died approximately 36–48 h in the experiment (T48, Fig. 4d).

### Superoxide dismutase 1 (SOD1) and catalase (CAT) detection

**Fig. 6:**
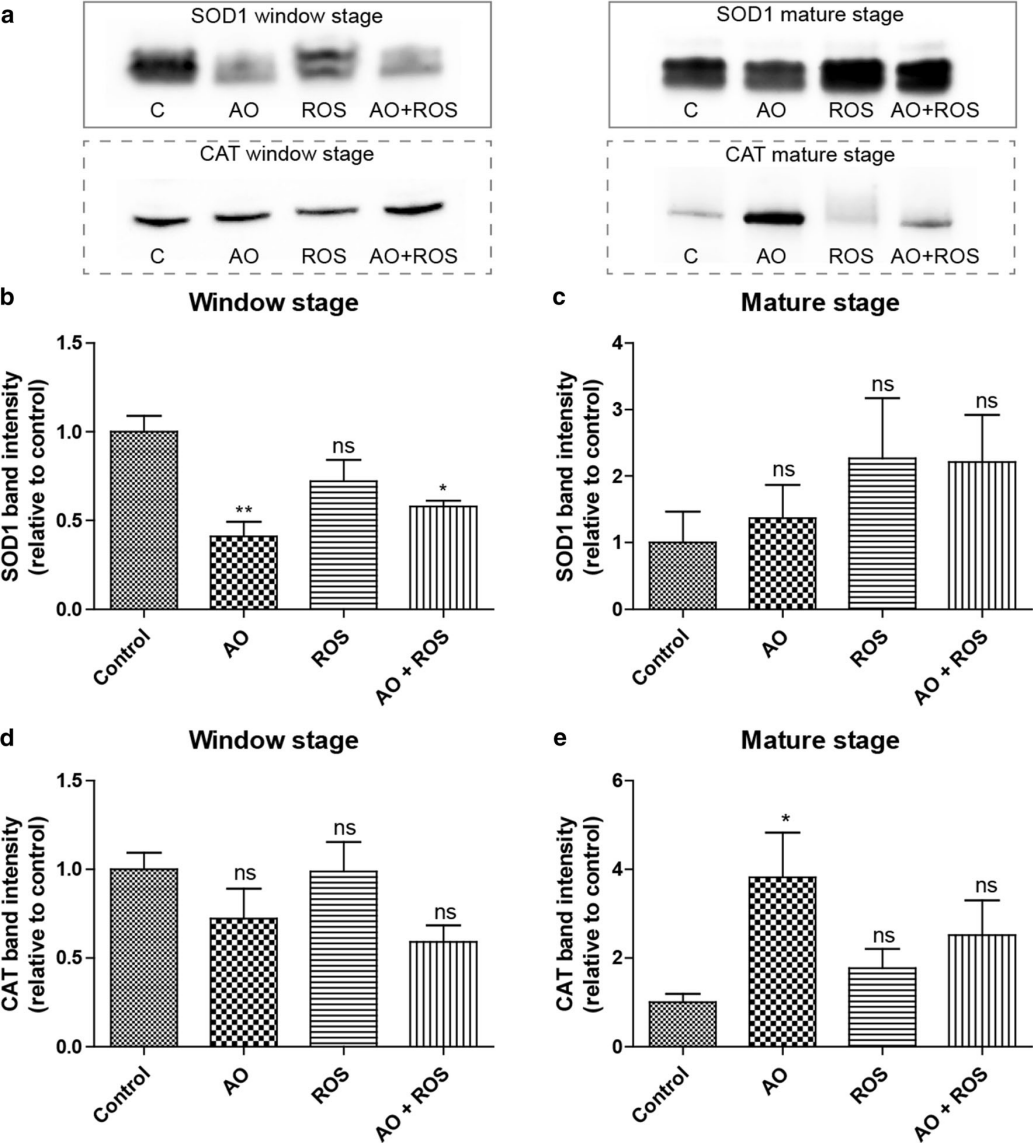
Detection of superoxide dismutase 1 (SOD1) and catalase (CAT) using western blotting for the control (C), antioxidant (AO; 400 µg/ml ascorbic acid and 200 µg/ml cysteine), 1 mM H_2_O_2_ reactive oxygen species (ROS), and AO + ROS treatment groups. Immunoprobing for SOD1 and CAT (**a**). SOD1 mean protein band intensities for window stage (**b**) and mature leaves (**c**). CAT mean protein band intensities for window stage (**d**) and mature leaves (**e**). One-way ANOVA, Dunnett’s multiple comparison test, **P < 0.01; *P < 0.05 ns, non-significant; *n* ≥ 4. *Error bars* represent standard error

The levels of two antioxidant enzymes (SOD1 and CAT) were investigated for window stage and mature leaf extracts via western blotting for the control and each of the treatments (Fig. 6). In general, window stage leaves had less SOD1 than mature leaves (Fig. 6a). Among the window stage samples, the relative SOD1 band intensities were significantly lower in antioxidant, as well as the antioxidant + ROS treated leaves compared to controls, but there were no differences observed from the ROS (1 mM H_2_O_2_) treatment (Fig. 6a, b). No significant differences in SOD1 were detected among the treatment groups for mature stage leaves (Fig. 6a, c). The overall CAT levels were not different between window and mature leaves (Fig. 6a). The relative CAT band intensities showed no significant difference in window stage leaves among the control and other treatment groups (Fig. 6a, d). In mature leaves, the antioxidant-treated leaves had significantly higher CAT proteins than the control, but there were no differences between the control and either the H_2_O_2_ (ROS) or antioxidant + ROS treatment groups (Fig. 6a, e).

### Anthocyanin and ABTS spectrophotometric assays

Spectrophotometric assays were used to determine anthocyanin and antioxidant scavenging levels in window stage and mature lace plant leaves (Table 1). Total anthocyanin content (cyanidin-3-rutinoside equivalents, C3RE) ranged from 0.34 to 3.29 mg/g. In both window and mature leaves, plants exposed to the antioxidant combination treatment had the lowest anthocyanin levels. Among the window stage, the antioxidant treatment was significantly different compared to all other groups. Mature leaves had significantly lower anthocyanins levels compared to control, H_2_O_2,_ and the antioxidant + H_2_O_2_ window stage leaves, but did not differ from the window stage antioxidant group. The ABTS assay showed higher radical scavenging activity in window stage leaf extracts compared to mature leaves across all treatment groups; however, no significant differences were observed among treatment groups within each developmental stage. Anti-radical scavenging capacity from the ABTS assay ranged from 1.69 to 1.88 mg vitamin C equivalents per gram.

**Table 1:** Anthocyanin and 2,2′azino-bis(3-ethylbenzothiazoline-6-sulfonic acid) (ABTS) spectrophotometric assays

	Anthocyanin		ABTS	
	mg C3RE/g	Grouping	mg VCE/g	Grouping
Window stage				
Control	2.58 ± 0.10	AB	1.87 ± 0.04	A
Antioxidants	0.34 ± 0.17	C	1.88 ± 0.05	A
ROS (H_2_O_2_)	3.29 ± 0.29	A	1.87 ± 0.01	A
Antioxidants + ROS	2.27 ± 0.32	B	1.86 ± 0.03	A
Mature stage				
Control	0.95 ± 0.11	C	1.70 ± 0.03	B
Antioxidants	0.70 ± 0.14	C	1.73 ± 0.03	B
ROS (H_2_O_2_)	1.22 ± 0.23	C	1.75 ± 0.05	B
Antioxidants + ROS	0.71 ± 0.11	C	1.69 ± 0.04	B

Anthocyanin content was measured for window stage and mature leaves for the control, antioxidant [400 µg/ml ascorbic acid (AA) + 200 µg/ml cysteine (Cys)], ROS (1 mM H_2_O_2_), and the antioxidant + ROS treatment groups. Anti-radical scavenging activity was determined using the ABTS assay. Anthocyanin levels were expressed in cyanidin-3-rutinoside equivalents (C3RE) and ABTS in vitamin C equivalents (VCE). Means with different letters differ significantly. One-way ANOVA, Tukey test (*P* < 0.05; *n* ≥ 4)

## Discussion

Programmed cell death is integral for the development and defense of plants and critical for their sessile lifestyle (Bozhkov and Lam 2011; van Doorn et al. 2011). Although there have been great advances in our understanding of plant PCD, the underlying mechanisms regulating the process are still being unraveled (van Doorn et al. 2011; Olvera-Carrillo et al. 2015; Van Durme and Nowack 2016). The lace plant has emerged as an excellent model for studying developmental PCD during leaf morphogenesis (Dauphinee and Gunawardena 2015). A striking feature of the lace plant is the presence of anthocyanins during PCD in early leaf development and their distribution pattern in the cell death gradient. This study investigated the role of antioxidants and ROS during developmental PCD in the novel lace plant model system.

Whole plant experimentation was used to evaluate the effects of antioxidants and ROS on perforation formation. A gradient of concentrations was tested for either ascorbic acid or cysteine, along with a combination of the two, to determine which was most effective at reducing the number of perforations. Ascorbic acid (also vitamin C, or ascorbate) has long been known for its antioxidant properties and exogenous applications have been shown to reduce oxidative damage (Smirnoff and Wheeler 2000; Arrigoni and De Tullio 2002). Examples include the ability to reduce insult from salt stress in tomato seedlings (Shalata and Neumann 2001), and the hypersensitivity of vitamin C-deficient mutants to ozone and UVB stress (Smirnoff and Wheeler 2000). Similar to ascorbic acid, cysteine is an antioxidant and it is the rate-limiting precursor for glutathione production (Lu 2013). Glutathione is critical for redox homeostasis as it is a component of the ascorbate–glutathione pathway that deals with the degradation of H_2_O_2_ (Szalai et al. 2009). Whole plant treatments revealed that the optimized combination antioxidant treatment (400 µg/ml ascorbic acid and 200 µg/ml cysteine) significantly decreases the formation of perforations and does not have a significant effect on leaf lengths, suggesting that growth was not adversely affected. The combination of ascorbic acid and cysteine was more effective at inhibiting perforations than either of the compounds alone (Fig. 2f). This may be due to the increased quantity of antioxidants in this combination group, or the additional cysteine that can increase glutathione levels (Lu 2013) and may have stabilized the ascorbic acid, which is a known effect of glutathione in aqueous solutions (Touitou et al. 1996). The ability of H_2_O_2_ to induce PCD and its implication within the signaling cascade has been established (Gechev and Hille 2005; Gechev et al. 2006; Gadjev et al. 2008). The 1 mM H_2_O_2_ treatment did not affect the number of perforations compared to the control group (Fig. 2). However, its application did increase lace plant PCD in the antioxidant + ROS treatment group, as evidenced by the increased number of perforations in leaves 2 and 3 (Fig. 2). Therefore, the application of 1 mM H_2_O_2_ was able to negate the inhibitory effect of the antioxidant treatment, which is consistent with the literature.

NBT staining of leaves from each stage of perforation development indicated that O_2_^−^ anions accumulate in PCD cells suggesting that dying cells are under oxidative stress. Oxidative stress occurs when there is an imbalance between antioxidants and oxidants in favor of the oxidants that leads to intracellular damage and can trigger cell death if the insult is severe enough (Sies 1997; Kacprzyk et al. 2011). Strong NBT staining was not observed in NPCD cells, indicating that they have lower stress levels than neighboring PCD cells. Furthermore, it suggests that NPCD cells, which have abundant anthocyanins, have greater antioxidant levels and may be able to neutralize ROS effectively compared to PCD stage cells. Superoxide radicals are primarily generated by the electron transport chain of mitochondria and the membrane-bound PSI electron acceptor found in chloroplast thylakoids (Bowler et al. 1992; Gill and Tuteja 2010). The previous studies in the lace plant have shown that chloroplast degradation occurs as cells transition to the later phases of PCD (Wright et al. 2009), and mitochondrial dysfunction and loss of membrane potential occurs during these later stages as well (Lord et al. 2011). It is well established that as ROS accumulate, there is further damage to mitochondria and a reduction in antioxidant defense (Lin and Beal 2006). The positive feedback loop of ROS production and intracellular damage may be responsible for the sharp contrast observed in NBT staining between the NPCD and PCD regions. The fact that antioxidant-treated leaves also had less NBT staining, specifically in the window stage (Fig. 2e), further supports this notion.

Long-term live cell imaging experiments showed a similar trend to the whole plant results. The antioxidant treatment reduced the rate of death in PCD cells and increased the lifespan of the detached leaf significantly compared to the control. In contrast, the 5 mM H_2_O_2_ treatment increased cell death rates and leaves expired faster than the control. In addition to the increased lifespan, the antioxidant treatment appeared to promote the formation of a phenolic ring within the center of the areole, which was faint or incomplete in the other treatment groups by comparison. Histochemical tests with Fluorol Yellow 088 suggest that the phenolic rings (Fig. 4b) contain suberin (data not shown). Mature lace plant leaves develop brown-colored rings of suberin at the edge of the perforation boundaries to protect against pathogens and nutrient loss (Gunawardena et al. 2007). Similarly, in wounded Arabidopsis leaves, PCD and the deposition of phenolic compounds serve to prevent the entry of pathogens (Cui et al. 2013). In Arabidopsis *bos1* mutants lacking the wound response, there is a ROS-associated runaway cell death process throughout the plant. The phenolic rings observed here in antioxidant-treated window stage leaves appear to form a protective barrier to isolate dying cells and are believed to serve the same purpose as in mature lace plant leaves.

Western blot analysis was performed to determine how the levels of two critical antioxidant enzymes (SOD1 and CAT) differed between window stage leaves actively undergoing PCD, and mature stage leaves where developmental PCD has halted in all experimental plants. In general, there were higher levels of SOD1 in mature leaves compared to the window stage. Mature leaves have fully developed chloroplasts in comparison to the window stage, which may account for this observed difference, since chloroplasts are known sources of O_2_^−^ (Mignolet-Spruyt et al. 2016). Further support comes from the NBT staining, which revealed O_2_^−^ throughout the mature leaves. There was a significant decrease in SOD1 levels in the antioxidant, as well as the antioxidant + ROS treated window stage leaves compared to the control, which suggests that these cells were less stressed from O_2_^−^ and, therefore, required lower levels of the protein to maintain homeostasis. CAT levels did not vary significantly compared to the control in the window stage and mature leaves, with the exception of the antioxidant treatment, where the level of CAT was significantly higher in mature leaves. Studies have shown that ascorbic acid, cysteine, and glutathione inhibit CAT activity (Foulkes and Lemberg 1948; Davisons et al. 1986), and in response to ascorbic acid treatment, cells increase expression of ascorbate peroxidase and *CAT* (Ondrej et al. 2010), which may account for the strong effect observed in the mature stage antioxidant treatment group.

The spectrophotometric assays revealed that anthocyanin levels were generally lower in mature leaves compared to the window stage. In window stage samples, there was significantly lower anthocyanin in the antioxidant treatment compared to the control and the highest levels observed were in the H_2_O_2_ samples. These results matched observations in window stage leaves from whole plant experiments and further support the notion that anthocyanins are also produced by plants in response to stress (Chalker-Scott 1999). Anthocyanins are naturally present in young lace plant leaves. The ABTS assay indicated that mature leaves have significantly lower anti-radical activity compared to window stage leaves, which coincides with lower anthocyanin levels.

Anthocyanins are water-soluble phenolic pigments with antioxidant properties that are involved in various stress responses and can be found in nearly all plant tissues (Chalker-Scott 1999; Liakopoulos et al. 2006; Tanaka et al. 2008). They are often located in epidermal cell layers in leaf tissues; however, in the lace plant, they are found in the mesophyll, which is also known to occur in genera including: *Syzygium*, *Rhododendron*, *Viburnum*, and *Mahonia* (Chalker-Scott, 1999). Anthocyanins are also known to accumulate in young tissues in a process known as juvenile reddening and increase in aging tissues prior to senescence, which is a form of PCD (Chalker-Scott 1999; Lee 2002; Thomas et al. 2003). In addition, anthocyanins provide tolerance to environmental stress induced by drought, wounding, chilling or freezing, UVB, and heavy metals, and they offer resistance to herbivory and pathogens (Gould 2004). To the best of our knowledge, however, the lace plant represents the only known association of anthocyanins with PCD in the early leaf development. Further research is underway to understand the specific forms of anthocyanins present and their potential roles relative to development and stress in lace plant leaves.

Pre-perforation stage leaves emerge from the corm with a complete vein pattern and an abundance of anthocyanins (Gunawardena et al. 2004). As leaves reach the window stage of development, the PCD gradient is established, but it is not currently known how this gradient is formed or what developmental cue triggers anthocyanin biosynthesis in these young leaves. Anthocyanins are typically synthesized within individual cells and are not known to travel long distances throughout the plant body (Landi et al. 2015). However, the anthocyanin precursors naringenin, dihydrokaempferol, and dihydroquercetin can move from shoots to roots from cell-to-cell in Arabidopsis (Buer et al. 2007). Moreover, flavonoids can travel from cotyledons to the root tip through the vascular tissue (Buer et al. 2007). It may be possible that the veins transport the signals for anthocyanin biosynthesis, or its precursors to the areoles or cells, but there is no supporting evidence in the lace plant except for the loss of anthocyanins centrally within areoles at the onset of PCD. Catalase is a sink for H_2_O_2_ in plants, which is produced in various stress responses including high light exposure. Vanderauwera et al. (2005) found that catalase-deficient Arabidopsis plants, following exposure to intense light, upregulate a transcriptional cluster responsible for anthocyanin regulation and biosynthesis. It may be possible that an initial increase in ROS, specifically H_2_O_2_, contributes to the establishment of the antioxidant gradient observed in window stage leaves. Our results indicated that PCD-inhibited mature leaves following antioxidant exposure also have high catalase protein levels compared to control condition. The signal responsible for the establishment and subsequent disappearance of anthocyanins during PCD remains unknown in the lace plant. We hypothesize that a gradient of anthocyanins, which is highest in the NPCD cells, offers resistance to PCD induction.

Future work will include the identification of the molecular and biochemical mechanisms controlling PCD, as well as the initial developmental stimuli leading to the observed decrease of anthocyanins and increase in ROS levels described here. Candidates include phytohormones such as ethylene, salicylic acid, and jasmonates, or even ROS themselves. Ethylene has been implicated in lace plant PCD signaling during perforation formation and senescence (Dauphinee et al. 2012; Rantong et al. 2015); however, the relationship between ethylene, antioxidants, and ROS remains unknown. Positive feedback loops are known to exist between various hormones, reactive nitrogen species, and ROS, which ultimately trigger downstream effectors such as nucleases and proteases that carry out PCD (Van Breusegem and Dat 2006). The links between anthocyanins and vein patterning also warrant investigation. Disrupting vein development with auxin inhibitors such as *N*-1-naphthylphthalamidic acid or auxinole is hypothesized to alter the pattern of anthocyanin deposition and perforation formation.

**Fig. 7:**
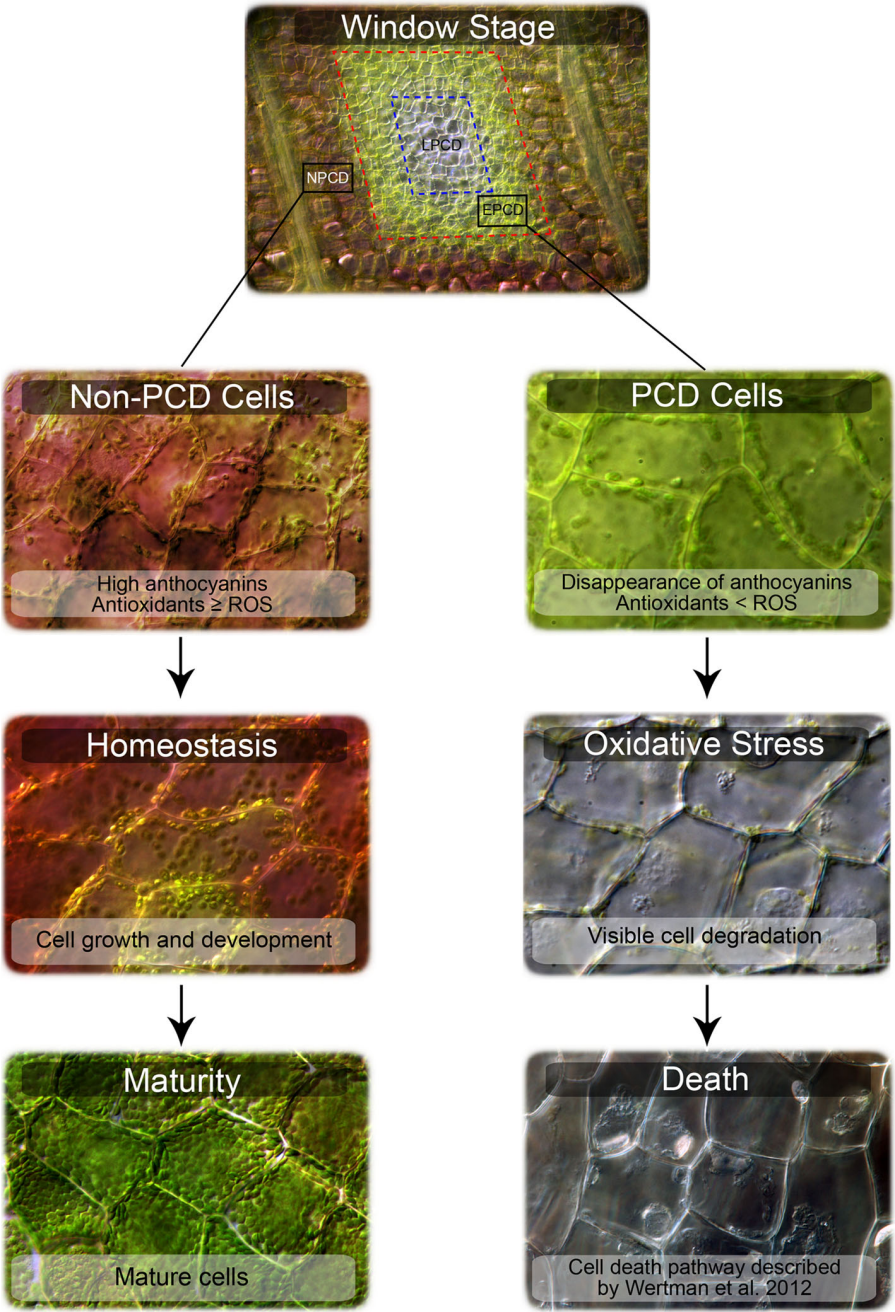
Antioxidants and reactive oxygen species (ROS) in lace plant developmental programmed cell death (PCD) signaling. The window stage of leaf development has a unique gradient of PCD. NPCD (non-PCD) cells within the gradient are found 4–5 cell layers from the veins and contain anthocyanins. Cells undergoing PCD are found centrally and have lost their anthocyanin pigmentation

A model and summary for the involvement of antioxidants and ROS in lace plant PCD are illustrated below (Fig. 7). Our results indicate that antioxidants are important for redox homeostasis and that the loss of antioxidants or increased ROS plays a significant role in the lace plant PCD pathway. Due to the rarity of this natural phenomenon and the conspicuous pattern of anthocyanins during PCD, we believe that investigation into the species of anthocyanins and antioxidants involved is a priority moving forward, as there may be potential industrial or medicinal applications for lace plant extracts.

### *Author contribution statement*

AND carried out all experimentation with the exception of the spectrophotometric assays. JIF ran the spectrophotometric assays and contributed to the whole plant experiments, plant culturing, and assisted with long-term live cell imaging. GLD contributed to the NBT staining and long-term live cell imaging. AND drafted the first MS including figure preparation and revised the final manuscript. AHLAN and CRL contributed to MS revisions and final MS preparation. AHLAN designed and supervised the experiments, while CRL co-supervised this study.

## Electronic supplementary material

Below is the link to the electronic supplementary material.

**ESM_1** Long-term live cell imaging of lace plant window stage leaf areoles. Leaves from the control, antioxidant, and 1 mM H_2_O_2_ and 5 mM H_2_O_2_ treatment groups were recorded for 12 h daily until the termination of all PCD cells. The sequence shown here reveals the views of entire areoles as cell death advanced over time (T). *Bar* = 100 µm (MP4 3562 kb)

**ESM_2** Long-term live cell imaging of lace plant window stage gradients. Leaves from the control, antioxidant, and 1 mM H_2_O_2_ and 5 mM H_2_O_2_ treatment groups were recorded for 12 h daily until the termination of all PCD cells. The sequence shown here reveals the progressions of cell death in window stage programmed cell death (PCD) gradient over time (T). *Bar* = 50 µm (MP4 2141 kb)

## Abbreviations

ABTS: 2,2′-Azino-bis-3-ethylbenzothiazoline-6-sulfonic acid
CAT: Catalase
NBT: Nitro blue tetrazolium
NPCD: Non-programmed cell death
PCD: Programmed cell death
ROS: Reactive oxygen species
SOD: Superoxide dismutase
